# Interactions between Auxin, Microtubules and XTHs Mediate Green Shade- Induced Petiole Elongation in Arabidopsis

**DOI:** 10.1371/journal.pone.0090587

**Published:** 2014-03-04

**Authors:** Rashmi Sasidharan, Diederik H. Keuskamp, Rik Kooke, Laurentius A. C. J. Voesenek, Ronald Pierik

**Affiliations:** 1 Plant Ecophysiology, Institute of Environmental Biology, Utrecht University, Utrecht, The Netherlands; 2 Environmental Sciences, Wageningen University, Wageningen, The Netherlands; 3 Laboratory of Plant Physiology, Wageningen University, Wageningen, The Netherlands; University of Texas at Austin, United States of America

## Abstract

Plants are highly attuned to translating environmental changes to appropriate modifications in growth. Such phenotypic plasticity is observed in dense vegetations, where shading by neighboring plants, triggers rapid unidirectional shoot growth (shade avoidance), such as petiole elongation, which is partly under the control of auxin. This growth is fuelled by cellular expansion requiring cell-wall modification by proteins such as xyloglucan endotransglucosylase/hydrolases (XTHs). Cortical microtubules (cMTs) are highly dynamic cytoskeletal structures that are also implicated in growth regulation. The objective of this study was to investigate the tripartite interaction between auxin, cMTs and XTHs in shade avoidance. Our results indicate a role for cMTs to control rapid petiole elongation in Arabidopsis during shade avoidance. Genetic and pharmacological perturbation of cMTs obliterated shade-induced growth and led to a reduction in XTH activity as well. Furthermore, the cMT disruption repressed the shade-induced expression of a specific set of *XTHs*. These *XTHs* were also regulated by the hormone auxin, an important regulator of plant developmental plasticity and also of several shade avoidance responses. Accordingly, the effect of cMT disruption on the shade enhanced *XTH* expression could be rescued by auxin application. Based on the results we hypothesize that cMTs can mediate petiole elongation during shade avoidance by regulating the expression of cell wall modifying proteins via control of auxin distribution.

## Introduction

The ‘shade avoidance syndrome’ (SAS), induced by aboveground plant-plant competition represents a classic example of successful adaptive environmental sensing and response [1], [2]. SAS is manifest in many plant species upon the detection of shade signals from neighbouring plants in crowded habitats and facilitates access to better lit, upper areas in a canopy. Leaves absorb light of specific wavelengths such as red and blue light, whereas others, such as far-red light, are reflected or transmitted [1]. The subsequent lowering of the red to far-red photon ratio (R:FR) is therefore an accurate and early indicator of neighbour proximity even in stages of vegetation development where leaf overlap and shading have not yet occurred [3]. When canopy closure occurs, shaded plants experience a simultaneous occurrence of both low R:FR and low blue. These reductions in blue light and R:FR are important cues that are sensed by the plant photoreceptors as a shading threat [3]–[5] and initiates a suite of morphological responses that constitutes the SAS. SAS includes enhanced shoot elongation, upward leaf movement (hyponasty), reduced apical dominance and acceleration of flowering [1], [2], [4], [6]. Shade-induced stem and petiole elongation involve primarily cellular expansion. Cellular expansion occurs when cell walls yield to turgor pressure within the cell resulting in a relaxation of wall stress. This ‘cell-wall loosening’ is the result of proteins that modify cell-wall structure [7], [8]. Two such protein families implicated in shade avoidance are expansins [9], [10] and xyloglucan endotransglucosylase/hydrolases (XTHs) [11], [12]. Upstream of the expansins and XTHs are various components regulating SAS. Amongst these is the phytohormone auxin, which is an important regulator of shade-induced growth responses in plants [13]. Shade cues result in an increase in both the biosynthesis and activity of auxin in elongating organs [14]–[18]. Furthermore, in Arabidopsis seedlings exposed to low R:FR, the auxin transporter protein PIN-FORMED3 (PIN3) changes from a basal to lateral distribution thus driving auxin towards the cortical and epidermal cells where cellular expansion occurs [19].

Cortical microtubules (cMTs) are highly dynamic structures that are important regulators of directional growth [20], [21]. cMT dynamics are influenced by environmental and hormonal factors and are therefore important sensors translating environmental cues to changes in plant growth [22], [23]. Although cMTs can be found in different patterns in the cell, most elongating cells display a transverse orientation (with respect to the long axis of the cell) [22], [24]. The disruption of these cMTs either using drugs [25] or due to a genetic mutation as seen in Arabidopsis mutants such as *microtubule organization1-1* (*mor1-1*) [26], *tonneau* [27] or *botero1* [28] leads to a loss of growth anisotropy and a characteristic swelling of the cells and organ. Similar effects are also seen when cellulose synthesis is inhibited using chemicals or in Arabidopsis cellulose deficient mutants such as *radially swollen1-1 (rsw1-1)* [29], [30], *korrigan (kor/rsw2)* [31], [32] and *kobito* [33] where the organization of the cellulose microfibrils (CMFs) is disrupted. Furthermore most elongating cells show a predominantly transverse orientation of both CMFs and cMTs. This coupled with studies demonstrating co-localization of cMTs and cellulose synthase complexes [34], has led to a model wherein cMTs control growth anisotropy by restricting cellulose deposition to certain parts of the cell wall, thereby constraining growth in one direction [20]. However, there have also been findings that demonstrated that this co-alignment could be uncoupled [25], [35]. It is now suggested that cMTs control the initial guidance of cellulose synthesizing complexes and subsequent CMF deposition could continue independently of cMTs. Regardless of the ambiguity of the cMT-CMF relationship, it is very well possible that other mechanisms exist via which cMTs could regulate the properties of the cell wall and therefore mediate growth regulation.

A recent study [36] has connected cMT orientation with the cellular localization of PIN proteins and thus polar auxin transport. Considering the important role of auxin and polar auxin transport in mediating shade avoidance responses, we investigated the role of cMTs in mediating shade-induced petiole elongation in Arabidopsis via regulation of auxin transport. Our results show that intact cMTs are needed for rapid unidirectional petiole growth during shade avoidance. When cMT organization is disrupted, the auxin redistribution that is required for the SAS is lost. We hypothesize that as a consequence of this, the *XTH* induction by shade is reduced as well.

## Results

### Shade avoidance responses in Arabidopsis require intact cMTs

**Figure 1:**
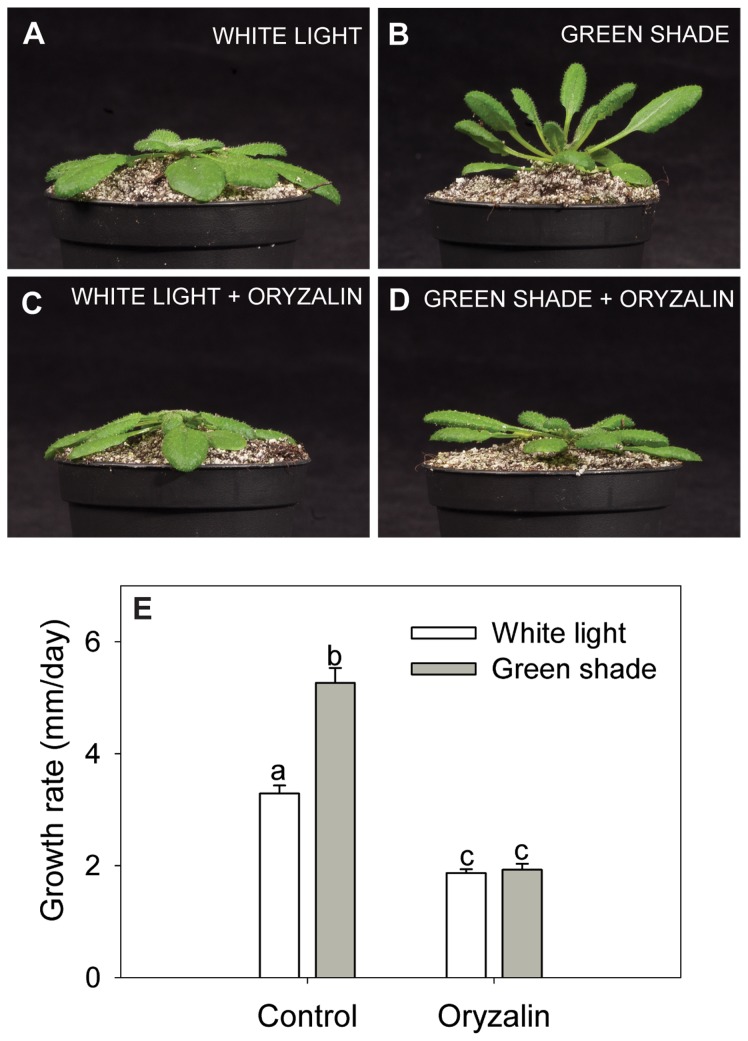
Shade avoidance in Arabidopsis requires intact cortical microtubules. (**A–D**) Arabidopsis (Col-0) plants after 24 h of white light (white bars) or green shade treatment with or without an oryzalin pre-treatment. (**E**) Petiole growth rates for Col-0 plants subjected to 24 h of control (white light; white bars) or green shade (gray bars) with or without oryzalin pre-treatment. Data points represent means ± SE (n = 10). Different letters above each bar indicate statistically significant differences (P<0.05, Tukey's *b* test).

Arabidopsis (Col-0) plants exposed to green shade for 24 h showed typical shade avoidance features (Figure 1A, 1B). Green shade is composed of reduced R:FR, reduced blue and reduced PAR; a realistic mimic of true shade underneath an overcast canopy. Shaded plants had petiole growth rates almost double that of control plants (Figure 1E) and displayed hyponastic leaves (Figure 1A, 1B). In order to determine the effect of cMT disruption on shade avoidance, plants were treated with the cMT disrupting drug oryzalin. Based on an earlier study [37] on plants at a similar developmental stage as used in this investigation and a dose-response assay (Figure S1), a thin film of oryzalin (200 µM) was applied to the petiole surface of rosette-stage plants that effectively disrupted cMTs without noticeable toxicity symptoms. Considering the mode of application and the presence of the cuticle as a barrier, we expect the effective concentration within the cells to be much lower than the applied concentration. Oryzalin pre-treated plants did not enhance petiole elongation upon green shade treatment. Petiole elongation rates in these plants were similar to untreated plants grown in shade (Figure 1E). Furthermore, disruption of cMTs also resulted in reduced shade-induced leaf hyponasty (Figure 1D). Genetic disruption of cMTs using the temperature sensitive *mor1-1* mutant showed similar results where the mutant displayed severely reduced shade-induced petiole elongation rates (Figure S2) validating the results of the pharmacological approach using oryzalin. MOR1 is a microtubule associated protein essential for CMT organization. *mor1-1* mutants display wild type characteristics at 20°C and transfer to the restrictive temperature of 30°C results in the disruption of only the CMT array [26].

### cMT disruption affects auxin activity and transport

**Figure 2:**
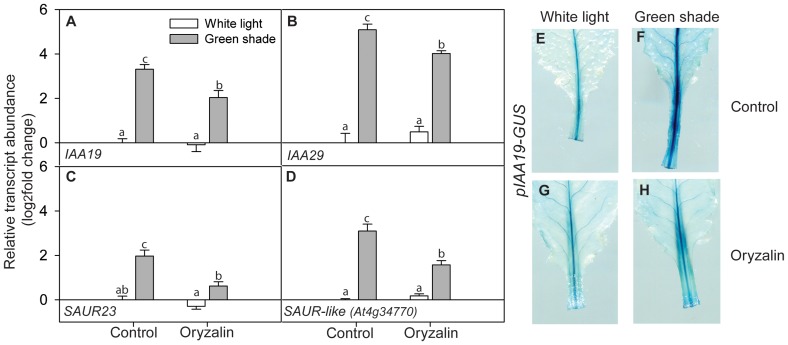
Perturbation of cortical microtubules affects auxin activity during shade avoidance. (**A–D**) Relative transcript abundance of selected auxin activity marker genes after 24 h of white light (white bars) or green shade (gray bars) treatment with or without oryzalin pre-treatment. Values are expressed on a log2 fold scale and were normalized using *AtUBQ10* as an internal control. Data points represent means ± SE (n = 5). Different letters above each bar indicate statistically significant differences (P<0.05, Tukey's *b* test). (**E–H**) Visualization of auxin activity in *pIAA19-GUS* transgenic lines after 24 h of white light or green shade treatment with or without oryzalin pre-treatment. Images are of leaves from representative plants processed to visualize GUS staining.

Since auxin is an important regulator of the shade avoidance response [14], [19] we measured the expression of several genes known to be auxin responsive (Figure 2). Indeed, their expression levels were induced upon green shade (Figure 2A–2D). The expression of *IAA19* was also visualized using the p*IAA19-GUS* reporter line where intense GUS staining was observed primarily in the elongating organ i.e. petiole under green shade conditions (Figure 2E–2H).

Interestingly, oryzalin treatment resulted in the repression of the green shade induced expression of these auxin-inducible genes (Figure 2A–2D), which was also visible as a decrease in the intensity of staining throughout the petioles in oryzalin treated *pIAA19-GUS* plants (Figure 2E–2H). These results showed that cMTs could modulate the level of auxin response, which in itself is crucial to the shade-induced elongation response.

### Auxin can modulate cMTs dynamics during shade avoidance

**Figure 3:**
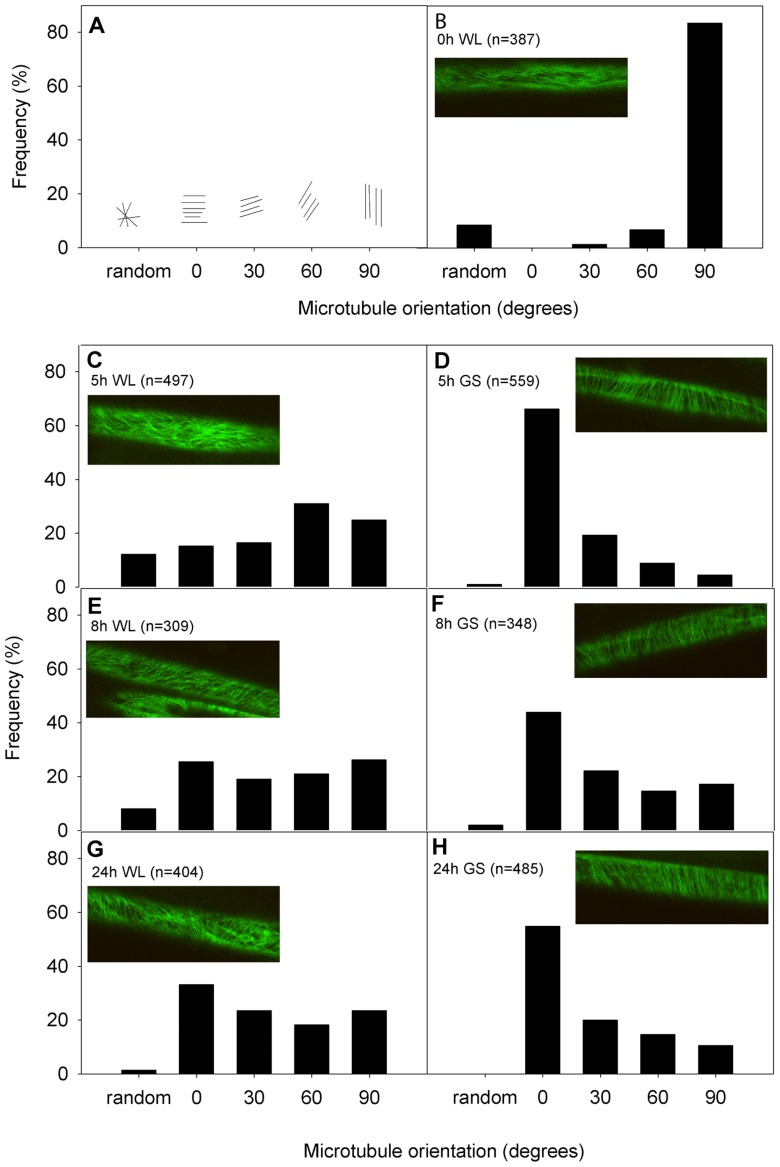
Cortical microtubules show typical orientation kinetics in response to shade. (**A**) The five microtubule orientation classes used to quantitate cortical microtubule dynamics in the petioles of Arabidopsis *GFP-TUA6* plants during shade avoidance. (**B–H**) Percentage cortical microtubules in different orientation classes at different time points of control (WL  =  white light) and shade (GS  =  green shade) treatment. Microtubule areas in the epidermal cells of petioles were scored at 0 h (B), 5 h (**C–D**), 8 h (**E–F**) and 24 h (**G–H**) after the start of treatment. At least 3–5 petioles per time-point per treatment were imaged. The total number of microtubule areas scored are indicated within each panel. Inset images are representative of a typical microtubule orientation for that treatment and time-point.

To visualize the orientation dynamics of cMTs in Arabidopsis petioles, *GFP-TUA6* plants were used. cMTs were imaged in cells of petioles from green shade and control plants and dynamics were quantified using five orientation classes (Figure 3A). At the start of the experiment more than 80% of the cMTs scored were in the 90°-orientation class (longitudinal to the growth axis) (Figure 3). At subsequent time points, starting from 5 h after treatment start, in green shade treatment, zero° (transverse to the growth axis) was always the predominant orientation class. In contrast, cMTs in petioles of control plants were more evenly distributed across the different orientation classes, gradually changing towards a high number of cMTs in the transverse orientation at t = 24 h (Figure 3C, 3G, 3E). Thus green shade promoted a faster transverse reorientation of cMTs in the petioles compared to white light controls.

**Figure 4:**
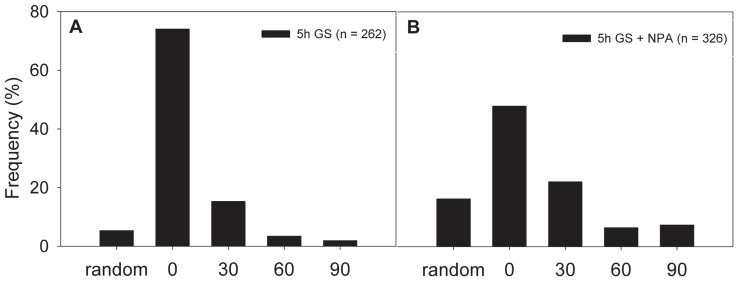
Auxin affects orientation of cortical microtubules during shade avoidance. (**A–B**) The effect of blocking polar auxin transport on the orientation behavior of cortical microtubules in the petioles of *GFP-TUA6* plants after 5 h of control (WL =  white light) and shade (GS =  green shade) treatment. At least 3–5 petioles per time-point per treatment were imaged. The total number of microtubule areas scored is indicated within each panel.

Although cMT integrity was found to affect auxin response genes (Figure 2), it is also known that auxin can affect cMT orientation [38]. The effect of auxin on green shade-mediated cMT orientation was experimentally investigated by treating *GFP-TUA6* plants with the auxin transport inhibitor, NPA (Figure 4). cMT orientations in the control and NPA treated petioles of plants from green shade were then quantitated. The percentage of transverse cMTs dropped from ∼74% in green shade treated petioles to ∼47% in petioles pre-treated with NPA and then exposed to green shade (t = 5 h). These data suggest that intact auxin transport is needed for the green shade induced change in cMT orientation, whereas at the same time auxin responses seem to be regulated by the cMTs as well (Figure 2).

### The interplay between cMTs and XTHs in the shade avoidance response of Arabidopsis petioles

**Figure 5:**
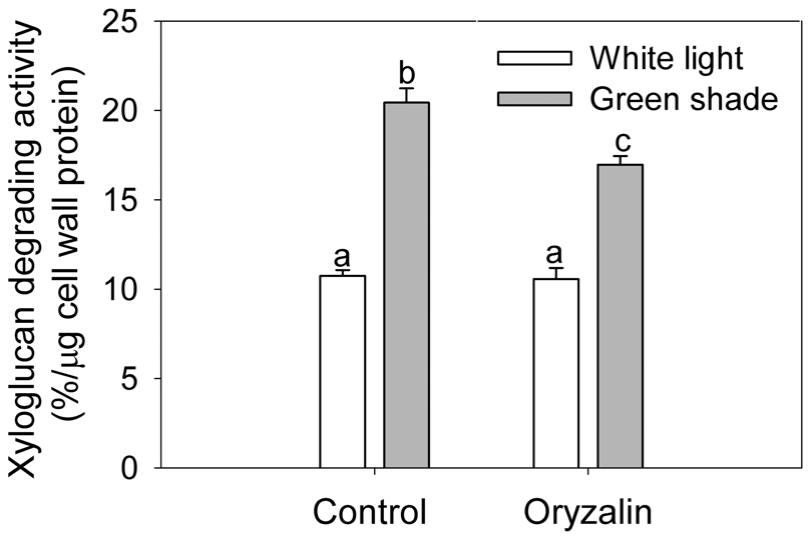
Perturbation of cortical microtubules affects XTH activity during shade avoidance. XTH activity measured as xyloglucan degrading activity in Col-0 petioles after 24 h of white light (white bars) or green shade (gray bar) treatment with or without oryzalin pre-treatment. Data points represent means ± SE (n = 4). Different letters above each bar indicate statistically significant differences (P<0.05, Tukey's *b* test).

XTHs are cell-wall loosening proteins that have been demonstrated to be functional to shade-induced petiole elongation in Arabidopsis [12]. In order to investigate whether the reduction of petiole growth rates upon cMT disruption was related to an effect on cell-wall modification activity, we measured XTH activity in Arabidopsis petioles. XTH activity was measured as Xyloglucan Degrading Activity (XDA) [10], [39] in Arabidopsis petioles (Figure 5). Petioles from green shade treated plants had significantly increased XDA relative to petioles from control plants. Treatment with oryzalin caused a significant reduction in XDA in the petioles of green shade treated plants compared to that in control plants (while under control light conditions it did not). Again, similar results were observed in the *mor1-1* mutant under conditions that disrupted cMT organization (Figure S3). Thus both chemical and genetic disruption of cMTs significantly reduced shade-induced increase in XDA.

**Figure 6:**
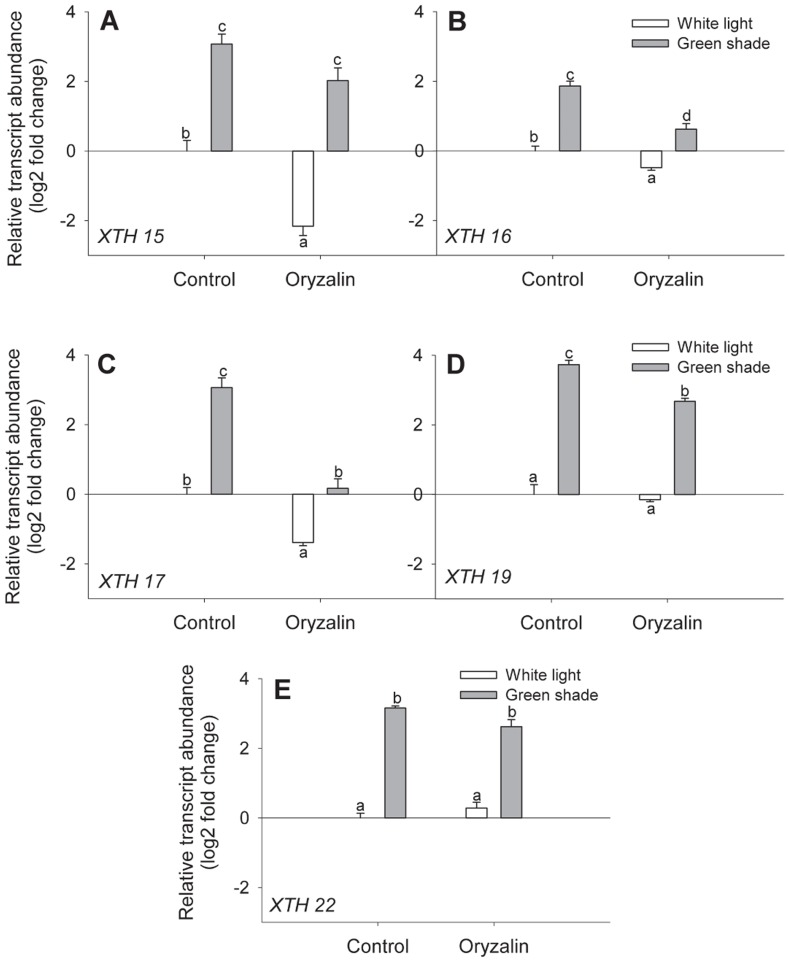
Perturbation of cortical microtubules affects *XTH* transcript abundance during shade avoidance. (**A–E**) Relative transcript abundance of five *XTH* genes in petioles of Col-0 plants after 24 h of white light (white bars) or green shade (gray bars) treatment with or without oryzalin pre-treatment. Values are expressed on a log2 fold scale and were normalized using *AtUBQ10* as an internal control. Data points represent means ± SE (n = 5). Different letters above each bar indicate statistically significant differences (P<0.05, Tukey's *b* test).

We also determined whether the observed reduction of XTH activity in petioles of oryzalin treated plants was the result of an effect on *XTH* gene expression. There are 33 *XTHs* in Arabidopsis, but previous work [12] has identified a specific subset of *XTH*s that are up regulated in the petioles of Arabidopsis plants at the same developmental stage as used here. We therefore tested this subset *(XTH 15, -16, -17, -19* and *-22)* and found them to be significantly up regulated in the petioles of green shade exposed plants relative to controls (Figure 6). However, in plants that were treated with oryzalin, the green shade-induced transcriptional induction of *XTH -16, -17* and -*19* was significantly reduced relative to their untreated green shade controls (Figure 6B–6D), whereas expression of *XTH15* and *XTH 22* was not affected by oryzalin. These data show that cMT disruption does not (only) affect the abundance of XTHs in the cell wall but also affects the expression of some *XTHs*. We next hypothesized that the interaction between cMTs and auxin as shown above might explain this effect, since auxin is known to regulate *XTH* expression during shade avoidance as well [18].

### Auxin regulates a specific subset of shade-induced *XTH* genes

**Figure 7:**
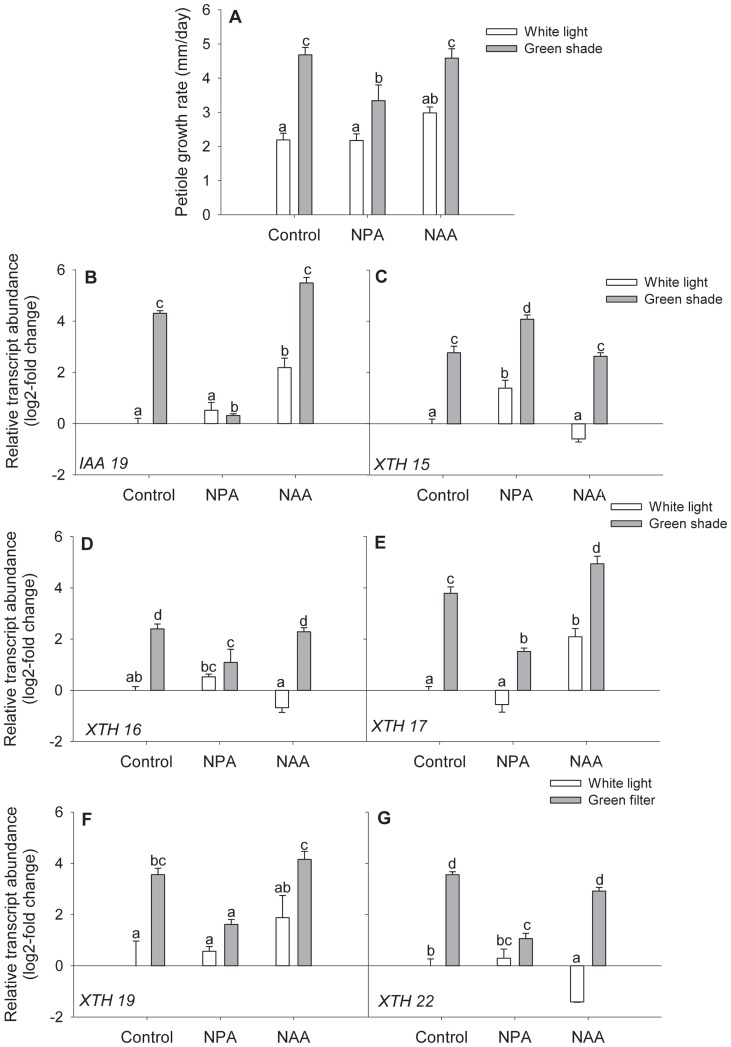
Auxin regulation of shade-induced *XTHs*. (**A**) Petiole growth rates of Col-0 plants after after 24 h of control (white bars) or green shade (gray bars) treatment with either an NPA or NAA pre-treatment. Data points represent means ± SE (n = 10). Different letters above each bar indicate statistically significant differences (P<0.05, Tukey's *b* test). (**B–G**) Relative transcript abundance of five green shade-induced *XTH* genes after 24 h of white light (white bars) or green shade (gray bars) treatment with either an NPA or NAA pre-treatment. Values are expressed on a log2 fold scale and were normalized using *AtUBQ10* as an internal control. Data points represent means ± SE (n = 5). Different letters above each bar indicate statistically significant differences (P<0.05, Tukey's *b* test).

**Figure 8:**
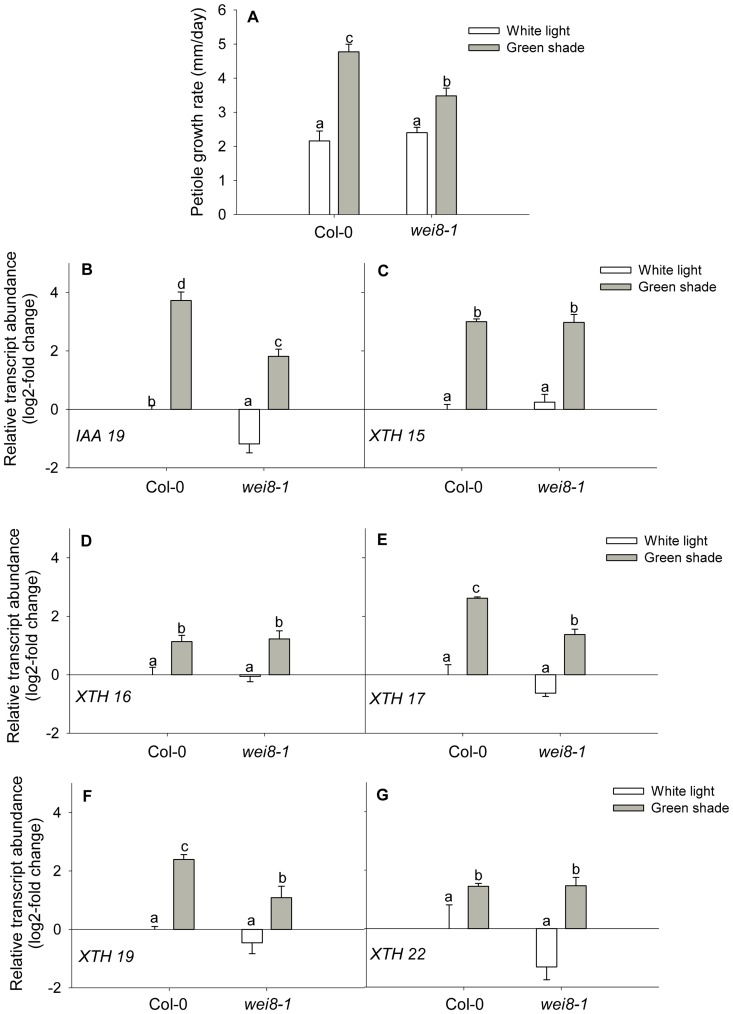
Differential regulation of green shade-induced *XTHs* in the auxin biosynthetic mutant *wei8-1*. (**A**) Petiole growth rates of wild-type (Col-0) and *wei8-1* plants after 24 h of white light (white bars) or green shade (gray bars) treatment. Data points represent means ± SE (n = 10). Different letters above each bar indicate statistically significant differences (P<0.05, Tukey's *b* test). (**B–G**) Relative transcript abundance of five green shade-induced *XTH* genes in the petioles of wild-type (Col-0) and *wei8-1* plants after 24 h of white light (white bars) or green shade (gray bars) treatment. Values are expressed on a log2 fold scale and were normalized using *AtUBQ10* as an internal control. Data points represent means ± SE (n = 5). Different letters above each bar indicate statistically significant differences (P<0.05, Tukey's *b* test).

To investigate the relation between CMTs, auxin and *XTH*s, we first determined whether there was an overlap between auxin regulated *XTHs* and those affected by oryzalin. Auxin regulation of *XTH* genes was determined by quantitating the expression of shade-induced *XTHs* in plants treated with the auxin transport inhibitor NPA and the synthetic auxin 1-naphthaleneacetic acid (NAA). NPA treatment significantly reduced but did not abolish petiole growth rates in green shade relative to untreated controls, indicating that polar auxin transport is at least partially required for this response (Figure 7A). NAA treatment stimulated petiole growth rates in white light grown plants compared to untreated controls (Figure 7A). Accordingly, NPA completely abolished the shade-induced up regulation of *IAA19*, whereas NAA treatment significantly increased its expression even in the absence of shade (Figure 7B). Out of the five green shade-responsive *XTHs*, NPA treatment significantly affected the green shade-induced differential expression of *XTH 16*, *XTH 17*, *XTH 19* and *XTH 22* (Figure 7D–7G). However, NAA application increased transcript levels of only *XTH 17* and *XTH 19* (Figure 7E, 7F). In order to further clarify whether *XTH 17* and *XTH 19* were the only two *XTHs* that were truly auxin regulated, we performed experiments with the *weak ethylene insensititve8-1* (*wei8-1*) mutant [40]. This mutant is defective in the TAA1-YUCCA pathway for tryptophan-derived auxin biosynthesis via indole-3-pyruvate and that is essential for shade avoidance [41]. Consequently, *wei8-1* showed reduced petiole growth rates in green shade relative to wild-type Col-0 plants (Figure 8A) and in this mutant, *IAA19* expression levels in green shade were significantly reduced compared to wild-type plants (Figure 8B). Petiole elongation in normal light conditions is unaffected in *wei8-1* and growth rates and morphology is similar to wild-type plants (Figure 8A). After 24 h of green shade treatment, transcript levels of *XTH 17* and *XTH 19* were significantly reduced in *wei8-1* plants compared to wild-type, whereas *XTH 15, XTH 16* and *XTH 22* were induced by green shade in both the genotypes (Figure 8C–8G). Thus, out of the subset of *XTHs* up regulated in response to green shade, *XTH 17* and *XTH 19* are auxin regulated, which are interestingly also the *XTH* genes whose expression is affected by the oryzalin treatment (Figure 6).

### Auxin application rescues the effect of cMT disruption on *XTH* gene expression

**Figure 9:**
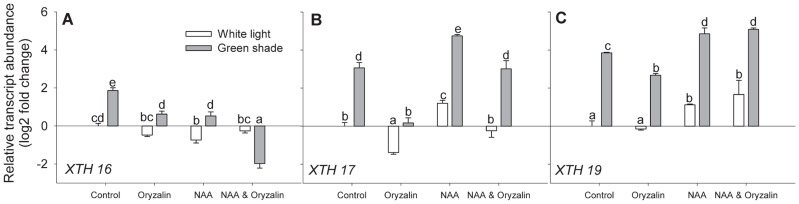
Auxin can rescue the effect of cortical microtubule disruption on XTH expression during shade avoidance. (**A–C**) Relative transcript abundance of three green shade-induced *XTH* genes that were also auxin regulated, after 24 h of white light (white bars) or green shade (gray bars) treatment with individual and combined oryzalin and NAA pre-treatments. Values are expressed on a log2 fold scale and were normalized using *AtUBQ10* as an internal control. Data points represent means ± SE (n = 5). Different letters above each bar indicate statistically significant differences (P<0.05, Tukey's *b* test).

If the disruption of cMTs affects *XTH* gene expression due to the effect on auxin re-distribution, then auxin application should be able to restore *XTH* transcript abundance to levels seen in untreated plants. This was investigated by applying auxin to oryzalin pre-treated plants before subjecting them to green shade conditions. Gene expression was measured for the three genes affected by oryzalin treatment *XTH 16, XTH 17* and *XTH 19* (Figure 6) out of which the latter two were also regulated by auxin (Figure 7–8). Figure 9 shows that the application of synthetic auxin NAA in combination with oryzalin resulted in the recovery of the green shade-induced up regulation of *XTH 17* and *XTH 19*, the two auxin regulated *XTHs*. These results suggest that indeed cMT orientation affects auxin transport, and not the auxin response itself, by which the *XTH* expression is regulated upon green shade, resulting in petiole elongation.

## Discussion

### Shade-induced growth and cMT reorientation

Light limitation due to crowding by neighbours is a frequently occurring stress in both natural plant distributions and agricultural fields. Rapid hyponastic growth and shoot elongation are early morphological responses of SAS and are both crucial to regain access to sunlight.

In Arabidopsis intact cMTs are critical to shade-induced petiole elongation and hyponasty (upward leaf movement), since cMT disruption abolished these responses in shade treated plants (Figure 1, S1 and S2). We are confident these effects were not a toxicity effect since first of all the plants did not show any visible or molecular toxicity symptoms and a similar application method and concentration was used by [37]. Secondly, genetic disruptions of cMT organisation by *mor1-1* resulted in the same defective shade-induced petiole elongation and XDA reduction (Figure S2 and S3). Thus intact cMT organization is essential for shade-induced petiole elongation.

cMTs are dynamic structures and can assume different orientations in a cell and undergo rapid shifts between these [22]. Shading caused typical changes in the organization of cMTs in the petioles of shade avoiding plants. Within just 5 h of green shade treatment, about 70% of the cMTs in the epidermal cells were oriented transversely (to the direction of growth) (Figure 3D). In contrast, the epidermal cells of petioles in white light maintained a mixed population of cMT orientations (Figure 3C, 3E, 3G). Orientation patterns of cMTs have been often considered indicators of the growth status of a cell. This is because expanding cells typically exhibit cMTs that are transversely oriented to the axis of growth and conversely non-elongating cells show cMTs parallel to the growth axis. The switch between different cMT orientations can be triggered by various endogenous and exogenous signals that are known to modulate growth, including light [38] and various hormones [42]. Studies in maize coleoptiles have previously demonstrated both phytochrome and blue light mediated cMT reorientations, possibly through control of certain phytohormones [38]. We found that blocking auxin transport affected green shade-induced cMT reorientation, with a drop in the percentage of transversely oriented cMTs (Figure 4). Shade signals increase auxin levels [14], [16], [17], [19] and it is possible that the observed shade-induced cMT reorientation is the result of this auxin increase since auxin can influence cMT orientation [43], [44]. The overall effect of different cMT orienting factors thus, appears to be complex and hierarchical. Shade avoidance itself involves more hormonal regulators in Arabidopsis than just auxin, including ethylene, gibberellic acid (GA) and brassinosteroids (BR) [18], [45]–[47] and all these phytohormones can influence cMT orientations [48]–[50]. This combined with the observation that an auxin transport inhibitor did not completely abolish green shade-induced cMT transverse orientations (Figure 4) and petiole elongation, suggests that these three hormones could also modulate cMT dynamics during shade avoidance. This is also in accordance with the fact that blocking auxin transport does not completely block the green shade petiole elongation response (Figure 7A). cMT orientation in response to external light cues therefore, is probably the result of physiological changes resulting from these external signals.

### cMTs and auxin-regulated cell wall modification

We found that cMT perturbation significantly reduced shade-induced increase in XTH activity (Figure 5). This could be explained by the fact that the up regulation of certain *XTH* genes (*XTH -16, -17* and *-19*) in the petioles of shade avoiding plants was significantly reduced in oryzalin treated plants (Figure 6B–6D). The effect of oryzalin on gene expression was not a toxicity effect of the drug, since expression of housekeeping genes and some *XTH* genes was unaffected (Figure 6). The lack of repression of the expression of certain shade-regulated *XTH* genes to oryzalin also explains why XTH activity is not completely abolished upon treatment of oryzalin (Figure 5) or in the *mor1-1* mutants at restrictive temperature (Figure S3).

Out of the subset of green shade-induced genes, two (*XTH -17* and -*19*) were auxin-regulated genes. It is not surprising that auxin does not regulate all shade-induced genes [18]. Blocking auxin transport and biosynthesis only partially blocked shade-induced petiole elongation (Figure 7A). This suggests that while auxin is important to the response it is not the sole regulator. Studies have suggested a role for both GA and BR in mediating elongation responses to shade cues [15], [18], [45], [47]. Furthermore, there is evidence of a crosstalk between the auxin and BR hormone signaling pathways and regulation of some *XTH* genes by both [18], [46]. For example, *XTH 16* regulation in response to blue light depletion involves combined auxin and BR action [18]. It is possible that the effect of oryzalin on *XTH 16* transcript levels is because cMT disruption affects endomembrane trafficking events that regulate BR signaling [51]. Although endomembrane trafficking events in plants primarily involve the actin network, cMTs are suggested to regulate related events in the cortical zone beneath the plasma membrane [51], [52]. Furthermore, data from cell-type specific transcript profiling of the Arabidopsis seedling [53], [54] shows that *XTH 17* and *XTH 19* (but not *XTH 16*) are enriched in shoot epidermal cells where auxin action is expected to be high during SAS (Figure S4). Our findings so far indicated that the effect of oryzalin on *XTH* transcript abundance was indeed primarily due to disruption of auxin transport. This hypothesis was supported by the observation that the inhibitory effect of oryzalin on the expression of *XTH 17* and -*19* could be overcome by applying a synthetic auxin NAA (Figure 9).

### cMTs and auxin transport

Although auxin modulates cMT dynamics, intact cMTs seem to be essential to normal auxin activity as well (Figure 2). Studies have shown a link between cMT orientation and cellular PIN localization [36], [52] providing a mechanism for the observed effects of oryzalin on auxin and *XTH* gene expression dynamics. Polar auxin transport determined by the re-distribution of PIN auxin efflux carriers is indeed critical for hypocotyl elongation in response to low R:FR and *pin3-3* mutants consequently display reduced fitness when grown together with wild-type plants in a dense canopy [19]. Similar to these observations, we found lateral re-localization of PIN3-GFP in the endodermal cells of Arabidopsis seedlings during green shade induced hypocotyl elongation (Figure S5). Oryzalin treatment severely reduced hypocotyl elongation in response to green shade and resulted in a disturbed PIN3-GFP distribution (Figure S5). Furthermore, shade-mediated increase in *PIN3* expression itself was not affected by oryzalin, indicating a specific effect on protein localization (Figure S6). These data suggest that cMTs could be important regulators of shade-induced changes in auxin distribution.

cMTs have traditionally been suggested to control directional growth by controlling cellulose deposition. Co-localization of cMTs with cellulose synthesizing protein complexes [34], has led to a model wherein cellulose fibers laid down in a similar orientation would restrict growth in one direction [55]. A recent study found that cell wall-plasma membrane connections that involve cellulose-based attachments were required for maintenance of PIN locations [56]. cMTs might thus control PIN distribution via their control of cellulose deposition and orientation. Furthermore, cMTs often transport numerous protein cargos and associations between cMTs and cell-wall modifying proteins [57] and protein activity has been reported [58]. It would therefore be possible that transversely oriented cMTs cause localized wall loosening via transport of cell wall modifying protein complexes to specific cell walls. As a result, auxin-induced cellular expansion would be restricted to one direction resulting in anisotropic growth.

## Conclusions

Based on our results we propose the following hypothesis (Figure S7): Shading triggers an increase in auxin biosynthesis [14] and changes in cMT dynamics. The changes in cMT orientation may facilitate auxin re-distribution to the lateral zones of the petiole, notably the epidermis, where auxin dependent control of elongation probably occurs. Here auxin could promote directional cell expansion by stimulating the expression of a set of *XTH* genes that are required for loosening the cell wall, thereby facilitating cell expansion. This expansion will result in petiole elongation as part of the SAS.

Future studies on the molecular components mediating the re-orientation and change in cMT dynamics and the resulting re-distribution of auxin in the elongating organ will provide further mechanistic insight into role of the cytoskeleton as an environmental sensor mediating timely morphological and physiological responses in plants.

## Methods

### Plant material and growth

All wild-type (Col-0, NASC), transgenic *pIAA19-GUS* [59] *PIN3-GFP* [60] and mutant *mor1-1* [26], *wei8-1* [40] Arabidopsis lines used in this study were in the *Arabidopsis thaliana* L. Heynh. Colombia-0 (Col-0) wild type background, except *GFP-TUA6* (Col (gl1) background) [61]. Seeds were sown on pots filled with a moist soil and perlite (1∶2, v/v) mixture followed by 4 day stratification in the dark at 4°C. The pots were then placed in a growth chamber (9 h photoperiod; 180 µmol m^−2^ s^−1^ photosynthetically active radiation (PAR)) for 4 d to facilitate seed germination. Seedlings were then transferred to 70 ml pots filled the same soil/perlite mixture above but supplemented with 0.14 mg MgOCaO (17%; Vitasol BV, Stolwijk, The Netherlands) and 0.14 mg slow-release fertilizer (Osmocote “plus mini”; Scotts Europe bv, Heerlen, The Netherlands) and nutrient solution (20 ml per pot) [62]. Pots containing seedlings were then placed on automatically watered irrigation mats in the growth chamber (9 h photoperiod; 180 µmol m^−2^ s^−1^ PAR) for four weeks.

For seedling growth, seeds were first surface sterilized for 10 minutes in a bleach:ethanol (2∶8; v/v) mixture followed by ethanol and water washes. Seeds were then individually sown (∼25 seeds per plate) on solid agar plates with 8 g L^−1^ agar and 0.22 g L^−1^ Murashige and Skoog (Duchefa Haarlem, The Netherlands). The plates were then stratified for 3 d in the dark at 4°C and then germinated in a growth chamber with 180 µmol m^−2^ s^−1^ PAR and 16 h photoperiod at 20°C. 45 h after germination plates were placed in a green shade treatment or control light conditions (defined above). After the light treatment seedlings were photographed and hypocotyl lengths measured from these images using the software Image J.

### Light and temperature treatments

Plants were used for experiments four weeks after transplant to individual pots. For every experiment ‘control’ conditions refer to plants grown in standard white light conditions in a climate controlled growth chamber with an unaltered spectral composition and a PAR of 180 µmol m^−2^ s^−1^. To mimic light conditions in a dense canopy (green shade), a green color filter (Lee 122 Fern Green) was added to the white light background. This reduced PAR to 65 µmol m^−2^ s^−1^, the R:FR to 0.19 and the blue light photon fluence rate to 2 µmol m^−2^ s^−1^. All experiments were started at 10 am.

The temperature sensitive mutant *mor1-1* was used for the genetic disruption of cMTs. MOR1 is a microtubule associated protein essential for cMT organization. *mor1-1* mutants display wild-type characteristics at 20°C and transfer to the restrictive temperature of 30°C results in the disruption of only the cMT array [26]. For experiments with *mor1-1* mutants, plants growing at the permissive temperature of 20°C were put into a growth cabinet (with similar growth conditions as mentioned above) at the restrictive temperature of 30°C all other growth conditions remaining identical. *mor1-1* plants were allowed to stay at the restrictive temperature for 24 h to allow disruption of cMTs before start of the green shade treatment.

### Chemical applications

The chemical oryzalin (Sigma, USA) was used to depolymerise cMTs. The concentration of oryzalin needed was determined using a dose-response curve (Figure S1) and is similar to that used in [37]. Petioles of four-week-old *GFP-TUA6* plants were brushed with an oryzalin solution (200 µM in water and 2% DMSO; containing 0.1% Tween 20) a day before the start of the treatment. This allowed disruption of cMTs prior to the start of the green shade treatment. Control plants were brushed with water containing equal volumes of DMSO and Tween 20. Control and oryzalin treated plants were then transferred to control and green shade growth conditions for 24 h. The chemical NPA was used to block polar auxin transport. NAA was used as a synthetic auxin. NAA and NPA (both 25 µM in 2% DMSO; containing 0.1% Tween 20) were applied to plants using a brush. NAA was applied just before the start and twice during the treatment that lasted 24 h. Control plants were brushed with a mock solution. For experiments with seedlings, 150 µl of an oryzalin stock solution was applied as a thin layer to the surface of the agar, 8 h before the light treatment started at a final concentration of 20 µM.

### Measurement of plant growth

Care was taken to choose similar sized plants. The same leaf in each pot was marked with a small paint dot and the corresponding petiole length was measured using a digital caliper at relevant time points at the start and duration of specific drug and light treatments. For each treatment petiole lengths were measured from at least ten individual plants. Measurements were made for three independent trials.

### XTH activity measurement

Petioles from control and treated plants were harvested, immediately frozen in liquid nitrogen and stored at −80°C until they were ready to be used. Enzyme extracts (from at least 20–30 pooled petioles per biological replicate) and XTH activity measurements were as described in [12]. These enzyme extracts were used for the measurement of xyloglucan degrading activity (XDA) which is the sum of both the transglycosylating and hydrolytic activity of XTHs [63]. XDA can also include the hydrolytic activity of non-specific endoglucanases. However, parallel assays run to detect the possible contribution of endoglucanase hydrolytic activity showed that this was negligible at the termination of the assay. XDA expressed in this study can therefore be taken as a measure of transglucosylating activity expressed as percentages of XDA per microgram of cell wall protein. Protein estimation was performed using the Bradford assay using a commercially available Bradford reagent (BioRad, Veenendaal, The Netherlands) [64]. Each experiment used at least three biological replicates. Experiments were repeated at least twice.

### Real time RT-PCR

Petioles were harvested from relevant Arabidopsis lines 24 h after different light, hormone and inhibitor treatments. Per plant the same petiole was harvested as used in other experiments. 8–10 petioles were pooled to form one biological replicate. After harvest, plant material was immediately frozen and stored at −80°C. Total RNA was extracted using the RNeasy plant mini kit (Qiagen Inc, USA) and reverse transcribed using random hexamers and Superscript III Reverse transcriptase (Invitrogen). The resulting cDNA was used as a template for Real-time RT-PCR using *AtUBQ10* and *AtEF1A* as internal standards (Table S1). The reaction contained (in a total volume of 20 µl) 10 µl of SYBR Green Supermix (Bio-Rad, no 170-8882), 50 ng cDNA (25 ng for 18S rRNA) and gene specific primers (Table S1). Results shown are using *AtUBQ10* as an internal reference gene. However, similar results were obtained with *AtEF1A*. The following program was used for all the genes tested: 3 min at 95°C followed by 40 cycles of 30 s at 95°C, 30 s at gene specific annealing temperature (Table S1), 60 s at 72°C. Primer design and annealing temperatures were optimized for each primer pair so as to result in specific amplification of the transcript of interest while avoiding the formation of primer dimers. The Ct value for each gene was normalized relative to the Ct value of the internal reference genes. Relative transcript levels were calculated using the comparative Ct method [65].

### Confocal imaging

***GFP-TUA6***: Leaves from four week old Arabidopsis *GFP-TUA6* plants growing in green shade and control light treatments were detached from the plant at specific time points (5 h, 8 h and 24 h) just before visualization with the confocal microscope. Different parts of the petiole were imaged using an inverted confocal laser scanning microscope (Zeiss CLSM Pascal, 40X C-apochromat objective), in order to visualize the cMTs in the epidermal cells. The excitation wavelength was 488 nm, and for GFP emission a 505–530 nm bandpath filter was used. Care was taken to ensure that petioles were not subjected to the laser beam for more than 5 minutes.

***PIN3-GFP***: Seedlings from green shade and control light treatments with or without oryzalin treatment were imaged using a confocal laser scanning microscope (Zeiss CLSM Pascal, 40X C-apochromat objective) with similar settings as above. Light treatments lasted 8 h.

### Quantitation of cMT orientation

Quantitation of the cMT orientation in the epidermal cells was done as described in [66]. Briefly, cMT orientations were classified into the following five groups (perpendicular to the growth axis): randomly oriented, transverse (0°), 30° oblique (30°), 60° oblique (60°) and longitudinal (90°). Only areas of cMTs that were twice as long as the cell width were included in the quantitation. For each treatment, at least 3 petioles from individual plants were imaged per time point. Experiments were repeated twice (with similar results in individual experiments) and the data pooled.

### GUS staining

Leaves from *pIAA19-GUS* plants subjected to different treatments were harvested and immediately placed in cold acetone followed by vacuum infiltration. The acetone was then replaced by staining buffer (0.1 M phosphate buffer) containing 100 mM ferrocyanide and ferricyanide, followed by vacuum infiltration. The staining buffer was then replaced with fresh staining buffer containing 1 mM 5-bromo-4-chloro-3-indolyl glucuronide (X-Gluc) followed by another round of vacuum infiltration till all the samples sank. The samples were then incubated at 37°C overnight. Staining was stopped by replacing the X-gluc with alcohol. The samples were then cleared by passing through a graded alcohol series after which they were photographed using an Epson flatbed scanner.

### Statistical analyses

For all growth rates and real-time RT-PCR measurements, treatments and their respective controls were analysed using a two-way ANOVA followed by Tukey's *b* test (SPSS V18).

### Accession numbers

Accession numbers for all genes used in this study are listed in Table S1.

## Supporting Information

Figure S1 **Oryzalin reduces shade-induced petiole elongation.** (**A**) The effect of increasing concentrations of oryzalin on petiole elongation rates in green shade (gray circles) and white light (white circles). Data points represent means ± SE (n = 10). Different oryzalin concentrations were applied to petioles before the start of the green shade treatment. (**B–C**) Cortical microtubules in the epidermal cells of *GFP-TUA6* petioles without (B) and with (C) 200 µm oryzalin treatment followed by green shade treatment (24 h).
(TIF)

Figure S2 **Shade-induced petiole elongation in the** ***mor1-1*** **mutant.** Petiole growth rates for Col-0 (wild-type) and *mor1-1* temperature sensitive mutant plants subjected to 24 h of control (white light; white bars) or green shade (gray bars) at (**A**) permissive (20°C) and (**B**) restrictive (30°C) temperatures. Data points represent means ± SE (n = 10). Different letters above each bar indicate statistically significant differences (P<0.05, Tukey's *b* test).
(TIF)

Figure S3 **XTH activity in the** ***mor1-1*** **mutant.** XTH activity measured as xyloglucan degrading activity in wild-type (Col-0) and *mor1-1* petioles after 24 h of control (white bars) or green shade (gray bars) at (**A**) permissive (20°C) and (**B**) restrictive (30°C) temperatures. Data points represent means ± SE (n = 4). Different letters above each bar indicate statistically significant differences (P<0.05, Tukey's *b* test).
(TIF)

Figure S4 **Cell-type specific expression of** ***XTH 16, -17*** **and -** ***19*** **.** Cell-type specific transcript abundance of *XTH* genes in Arabidopsis shoots. Abundance of *XTH 16, 17* and *19* based on the amount of these transcripts associated with ribosomes. Data is based on the cell type-specific expression lines and data for control conditions described in Mustroph et al., 2009 and obtained from the online cell type specific eFP translatome browser (http://efp.ucr.edu). Images also indicate the regions in the shoot where the cell type specific promoters are expressed.
(TIF)

Figure S5 **Polar auxin transport during shade avoidance is disturbed by disruption of cortical microtubules.** (**A**) Hypocotyl lengths of Col-0 seedlings after 3 d of control (white bars) or green shade (gray bar) treatment with or without oryzalin pre-treatment. Data points represent means ± SE (n = 30–60). Different letters above each bar indicate statistically significant differences (P<0.05, Tukey's *b* test). (**B–D**) Confocal images of the hypocotyls of *PIN3-GFP* seedlings after 3 d of control (B) or green shade (C) treatment and green shade with an oryzalin pre-treatment (D). Images are representative of at least 5 seedlings that were imaged per treatment from 2 independent trials.
(TIF)

Figure S6 **Relative transcript abundance of** ***AtPIN3*** **in the petioles of white light or green shade treated plants with (black bars) or without (white bars) oryzalin treatment.** Data points represent means ± SE (n = 3–4). There were no significant differences between control and oryzalin treated samples (P<0.05, students t test).
(TIF)

Figure S7 **Tripartite interactions between auxin, XTHs and cortical microtubules in the shade avoidance response in Arabidopsis.**
(TIF)

Table S1 **Primers used for quantitative RT-PCR.**
(DOCX)
