# Supplementary figures and images for: Interactions between Auxin, Microtubules and XTHs Mediate Green Shade- Induced Petiole Elongation in Arabidopsis
Source: PLoS One. 2014 Mar 4;9(3):e90587. doi: 10.1371/journal.pone.0090587 (PMC3942468; doi:10.1371/journal.pone.0090587)

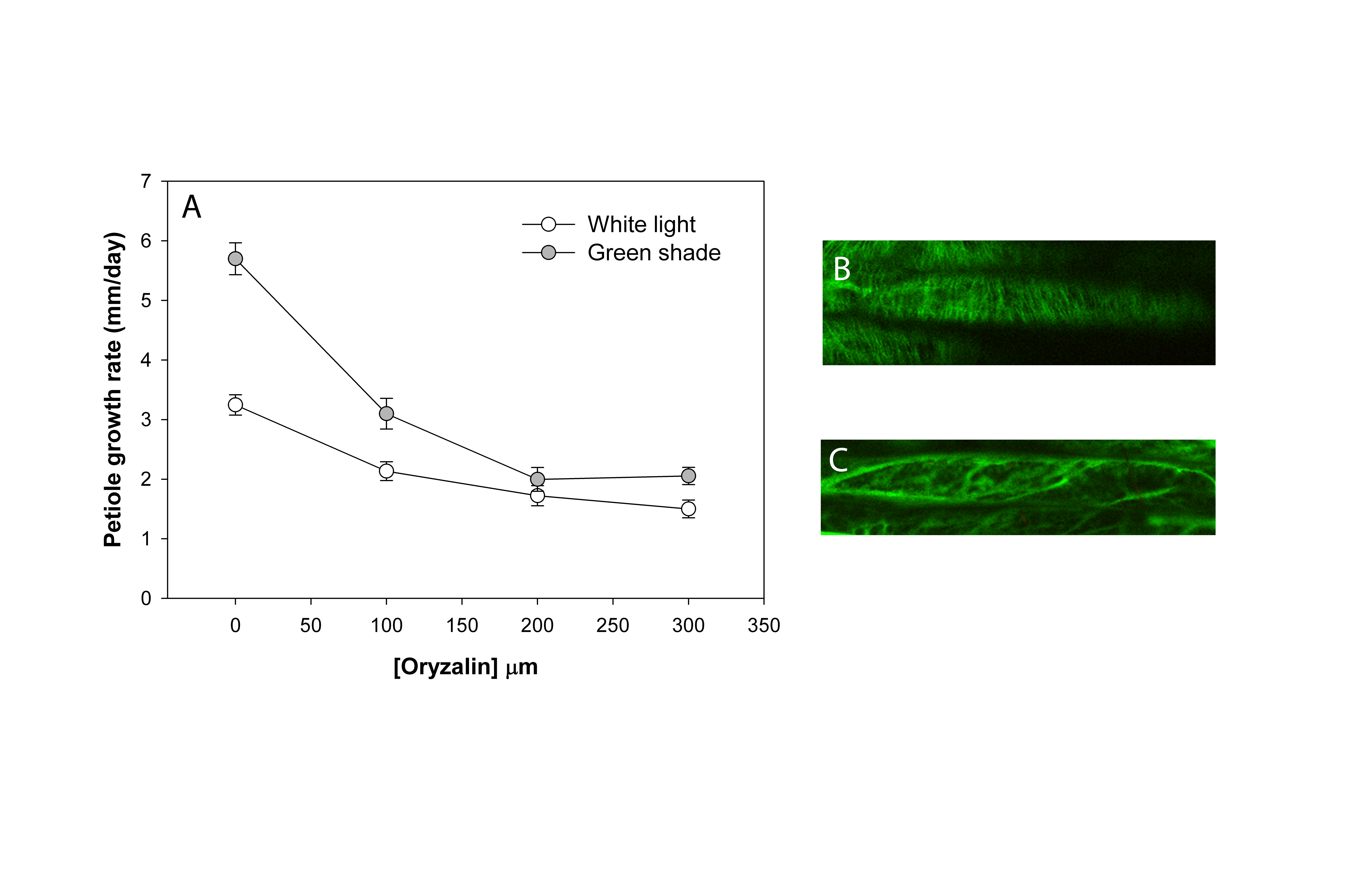

Supplement: Figure S1 — Oryzalin reduces shade-induced petiole elongation. (A) The effect of increasing concentrations of oryzalin on petiole elongation rates in green shade (gray circles) and white light (white circles). Data points represent means ± SE (n = 10). Different oryzalin concentrations were applied to petioles before the start of the green shade treatment. (B–C) Cortical microtubules in the epidermal cells of GFP-TUA6 petioles without (B) and with (C) 200 µm oryzalin treatment followed by green shade treatment (24 h). (TIF) [file pone.0090587.s001.tif]

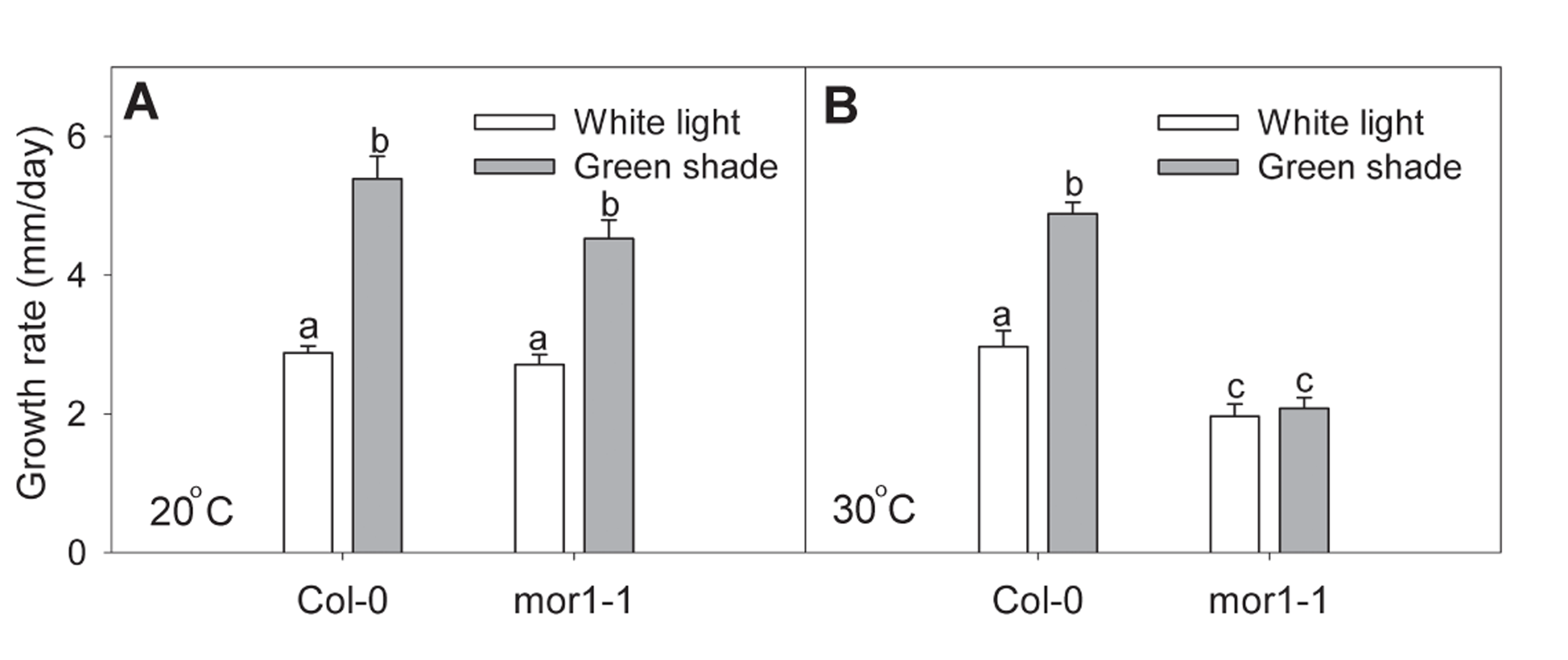

Supplement: Figure S2 — Shade-induced petiole elongation in the mor1-1 mutant. Petiole growth rates for Col-0 (wild-type) and mor1-1 temperature sensitive mutant plants subjected to 24 h of control (white light; white bars) or green shade (gray bars) at (A) permissive (20°C) and (B) restrictive (30°C) temperatures. Data points represent means ± SE (n = 10). Different letters above each bar indicate statistically significant differences (P<0.05, Tukey's b test). (TIF) [file pone.0090587.s002.tif]

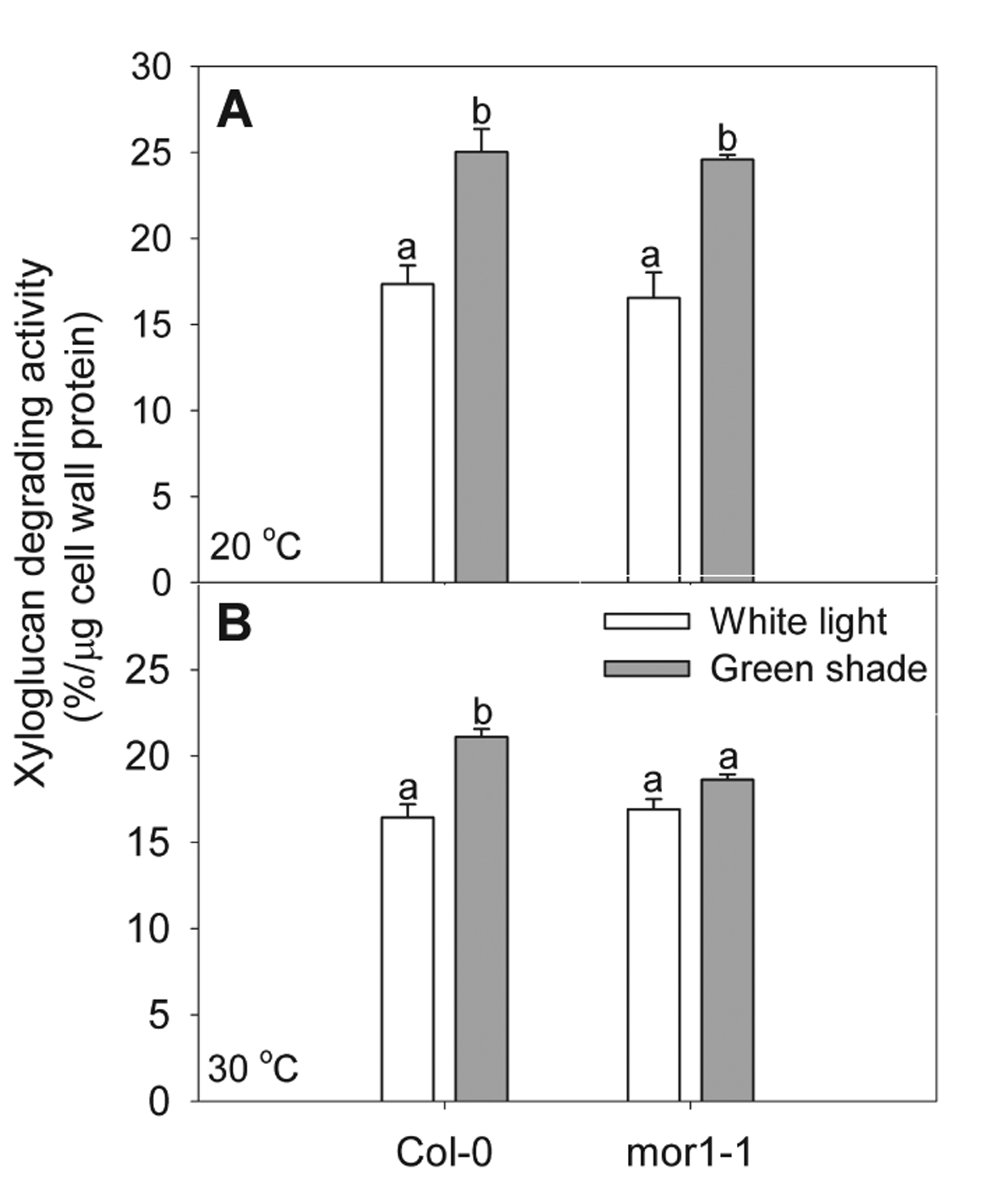

Supplement: Figure S3 — XTH activity in the mor1-1 mutant. XTH activity measured as xyloglucan degrading activity in wild-type (Col-0) and mor1-1 petioles after 24 h of control (white bars) or green shade (gray bars) at (A) permissive (20°C) and (B) restrictive (30°C) temperatures. Data points represent means ± SE (n = 4). Different letters above each bar indicate statistically significant differences (P<0.05, Tukey's b test). (TIF) [file pone.0090587.s003.tif]

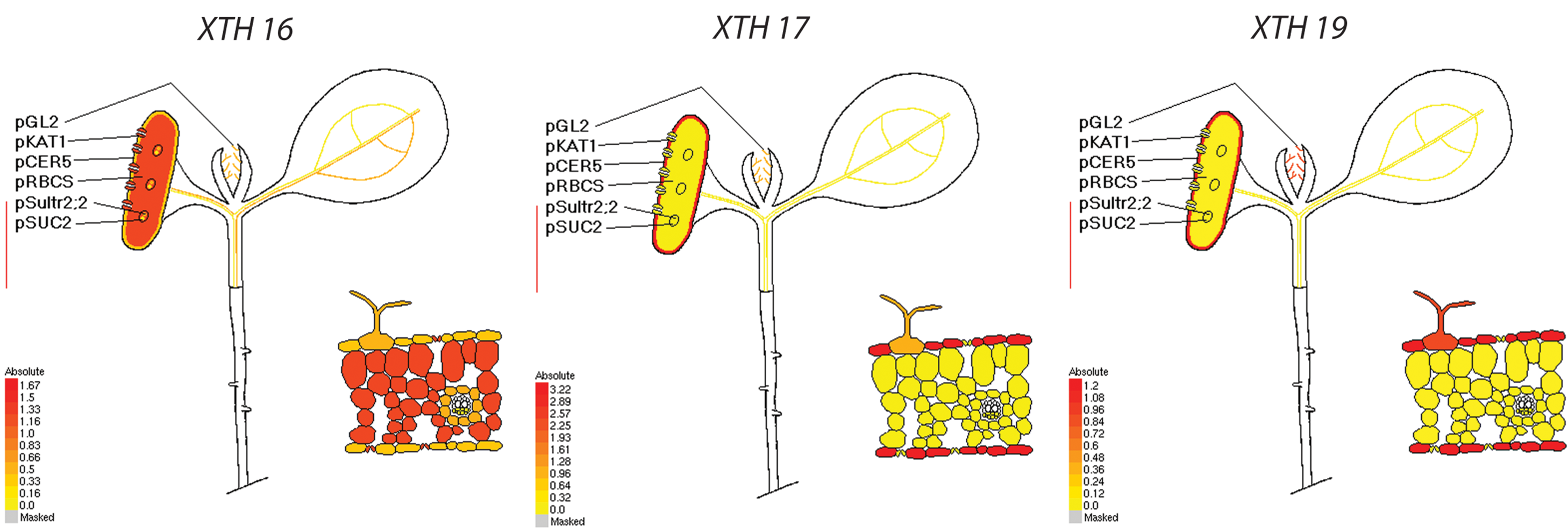

Supplement: Figure S4 — Cell-type specific expression of XTH 16, -17 and - 19 . Cell-type specific transcript abundance of XTH genes in Arabidopsis shoots. Abundance of XTH 16, 17 and 19 based on the amount of these transcripts associated with ribosomes. Data is based on the cell type-specific expression lines and data for control conditions described in Mustroph et al., 2009 and obtained from the online cell type specific eFP translatome browser (http://efp.ucr.edu). Images also indicate the regions in the shoot where the cell type specific promoters are expressed. (TIF) [file pone.0090587.s004.tif]

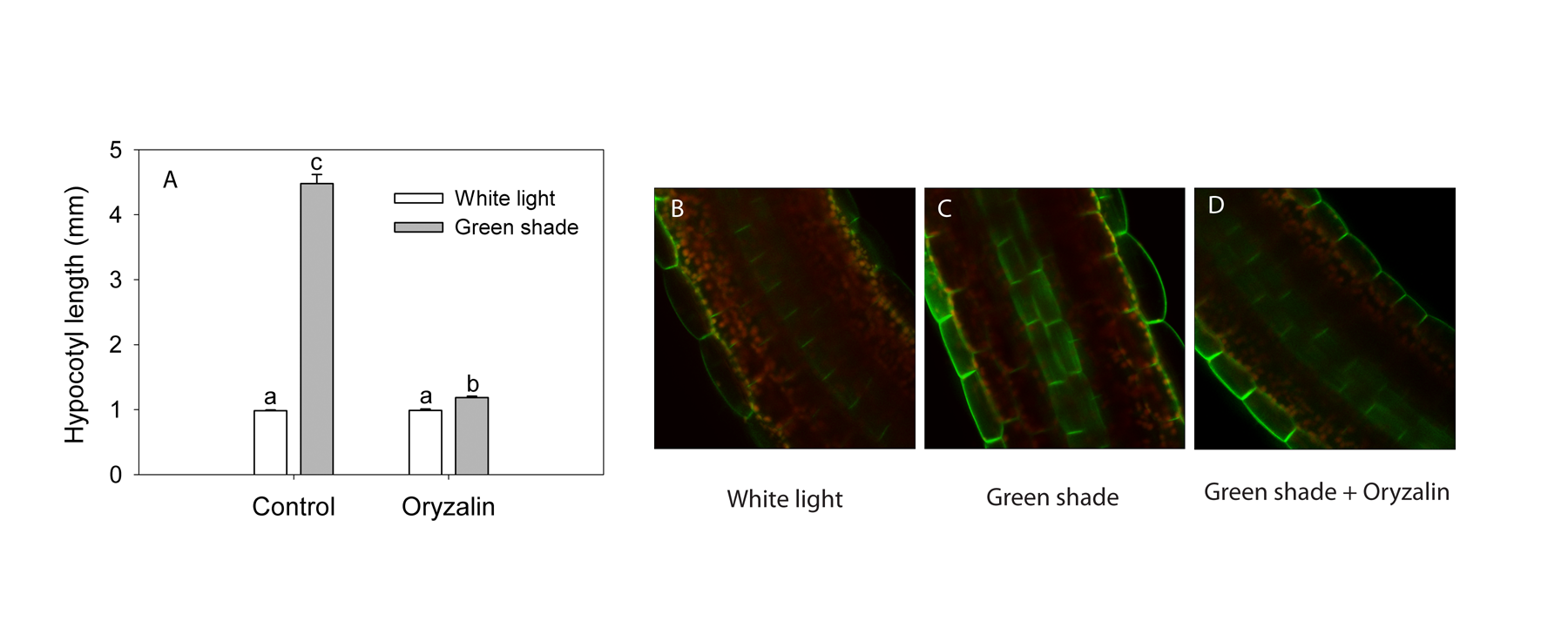

Supplement: Figure S5 — Polar auxin transport during shade avoidance is disturbed by disruption of cortical microtubules. (A) Hypocotyl lengths of Col-0 seedlings after 3 d of control (white bars) or green shade (gray bar) treatment with or without oryzalin pre-treatment. Data points represent means ± SE (n = 30–60). Different letters above each bar indicate statistically significant differences (P<0.05, Tukey's b test). (B–D) Confocal images of the hypocotyls of PIN3-GFP seedlings after 3 d of control (B) or green shade (C) treatment and green shade with an oryzalin pre-treatment (D). Images are representative of at least 5 seedlings that were imaged per treatment from 2 independent trials. (TIF) [file pone.0090587.s005.tif]

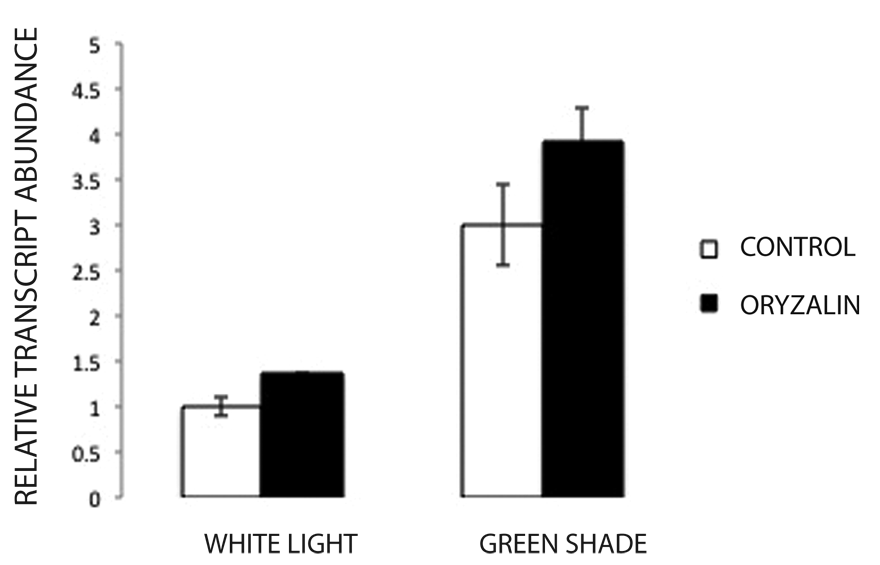

Supplement: Figure S6 — Relative transcript abundance of AtPIN3 in the petioles of white light or green shade treated plants with (black bars) or without (white bars) oryzalin treatment. Data points represent means ± SE (n = 3–4). There were no significant differences between control and oryzalin treated samples (P<0.05, students t test). (TIF) [file pone.0090587.s006.tif]

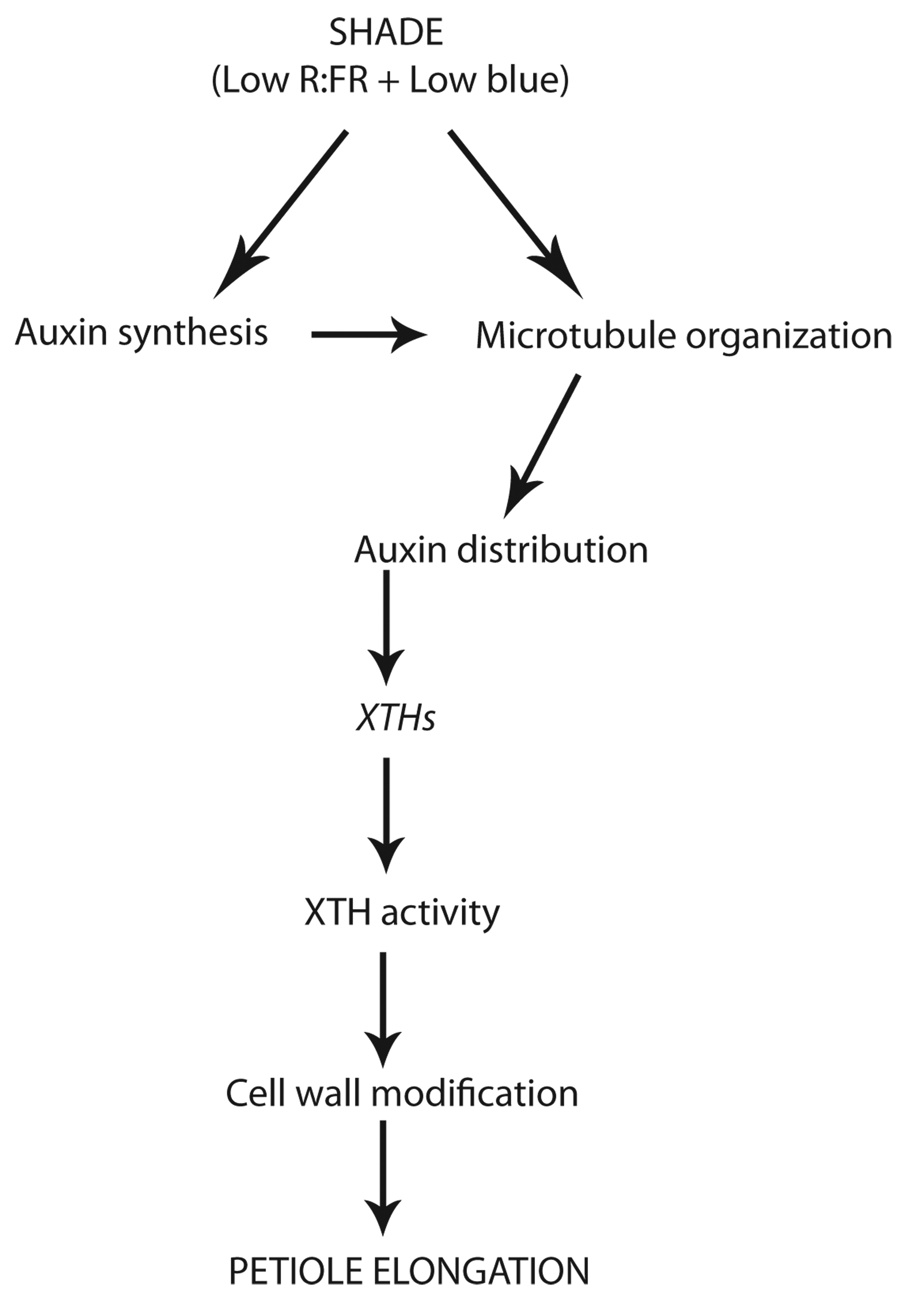

Supplement: Figure S7 — Tripartite interactions between auxin, XTHs and cortical microtubules in the shade avoidance response in Arabidopsis. (TIF) [file pone.0090587.s007.tif]
