# Supplementary material for: Interactions between Auxin, Microtubules and XTHs Mediate Green Shade- Induced Petiole Elongation in Arabidopsis
Source: PLoS One. 2014 Mar 4;9(3):e90587. doi: 10.1371/journal.pone.0090587 (PMC3942468; doi:10.1371/journal.pone.0090587)
Supplement: Table S1 — Primers used for quantitative RT-PCR. (DOCX) [file pone.0090587.s008.docx]

| **Gene name** | **AGI ID** | **Forward primer** | **Reverse primer** |
| --- | --- | --- | --- |
| *At3g15540* | *AtIAA19* | GGCTTGAGATAACGGAGCTG | accatctttcaaggccacac |
| *At4g14130* | *AtXTH15* | CGGCACCGTCACTGCTTAC | GAAACTCAAAGTCTATCTCGTCATGTG |
| *At3g23730* | *AtXTH16* | CCGGTAACTCCGCTGGAA | TCTCGTCGTGTGTTGGTCCTT |
| *At1g65310* | *AtXTH17* | atgggctaatggaaaatcatcttgtt | tactttgcacacctttcattcttgtc |
| *At4g30290* | *AtXTH19* | tgcagctaaatgattgattctttgat | ccattgagttacaaagacaacgtca |
| *At5g57560* | *AtXTH22* | ctaaagagtgcttagctgcatagagaga | caaatcaataaaattcacgtgatctacaa |
| *At5g18060* | *AtSAUR23* | CGTAGGAGAGAGCCAGAAGAAG | GGATAATCATCAATGGAGCCGAG |
| *At4g32280* | *AtIAA29* | ATCACCATCATTGCCCGTAT | ATTGCCACACCATCCATCTT |
| *At4g34770* | *AtSAUR-like* | GTCATGTGGCCGTCTATGTG | TCGAACCCGAACTCTTCTTC |
| *At4g05320* | *AtUBQ10* | ggccttgtataatccctgatgaataag | aaagagataacaggaacggaaacatagt |
| *At1g70940* | *AtPIN3* | GCGTCAATAAAAACCCGAAA | GGCGTCTTTTGGTCTCTCTG |
| *At5g60390* | *AtEF1A* | tccagctaagggtgcc | ggtgggtactcggaga |

**Table S1.** Primer sequences used for real-time PCR analyses.
